# An Unusual Presentation of Pseudothrombotic Microangiopathy in a Patient with Autoimmune Atrophic Gastritis

**DOI:** 10.1155/2016/1087831

**Published:** 2016-02-25

**Authors:** Alexandre Malek, Roy Nasnas

**Affiliations:** Infectious Diseases Department, Hotel Dieu De France Hospital, Faculty of Medicine, Saint Joseph University, Beyrouth, Lebanon

## Abstract

*Introduction*. We hereby describe the case of a young female patient who presented with pseudothrombotic microangiopathy, as well as pancytopenia accompanied by autoimmune atrophic gastritis.* Case Presentation*. A 36-year-old Caucasian woman presented to the emergency department with fatigue and dyspnea on minimal exertion. Physical examination was unremarkable except for pallor and noninjected conjunctiva. Laboratory tests revealed high LDH and low hemoglobin, white blood cells, platelets, and haptoglobin. The peripheral blood smear showed schistocytes suggestive of pseudothrombotic microangiopathy. Low cobalamin level and hyperhomocysteinemia were also detected. Autoimmune atrophic gastritis was confirmed by gastric biopsy and positive anti-intrinsic factor antibodies. Vitamin B12 supplements were given which led to rapid recovery and normalization of blood parameters.* Conclusion*. This case highlights the importance and serves as a reminder to clinicians to rule out cobalamin deficiency and autoimmune atrophic gastritis in patients presenting with a picture suggestive of thrombotic thrombocytopenic purpura and pancytopenia, which was completely reversible after appropriate replacement therapy without recurring to unnecessary and invasive procedures such as plasma exchange.

## 1. Introduction

Pseudothrombotic microangiopathy is a life-threatening hematological condition that has been described in very few cases of cobalamin deficiency. To date, it is a rare clinical entity, not well documented in medical practice, and more often it is misdiagnosed with other diseases. The cardinal clinical characteristics include dyspnea, asthenia, mild peripheral sensitive neuropathy, thrombocytopenia, and hemolytic anemia associated with numerous schistocytes [1].

Schistocytes are fragmented parts of red blood cells due to the circulation of blood through damaged capillaries and loss of endothelial system homeostasis. They are pathognomonic features of microangiopathic hemolytic anemia which is seen in thrombotic thrombocytopenic purpura (TTP)/hemolytic uremic syndrome (HUS), disseminated intravascular coagulation (DIC), bone metastatic cancers [2], and drug mediated thrombotic microangiopathy.

As for atrophic gastritis, it is a chronic inflammation of gastric mucosa that is characterized by progressive loss of gastric glandular cells. It is classified into two types: type A or autoimmune gastritis, which is an immune-mediated inflammation leading to vitamin B12 deficiency and depicting a systemic disease named pernicious anemia, whereas type B is a nonautoimmune gastritis [3].

Vitamin B12 is a cofactor for two important enzymes: methionine synthase and L-methylmalonyl coenzyme A mutase; both enzymes play an essential role in cellular DNA synthesis and neurologic function. Therefore, vitamin B12 deficiency can consequently lead to several clinical presentations like bone marrow failure, demyelinating nervous system disease, and neuropsychiatric manifestations [4].

In this paper, we describe the clinical manifestation, evolution, and treatment of an atypical case of pseudothrombotic microangiopathy in a young female patient secondary to autoimmune atrophic gastritis. In addition, the particularity of this case resides in the presence of pancytopenia, hemolytic anemia, elevated D-dimers, and numerous schistocytes on peripheral blood that might be mistaken for life-threatening thrombotic thrombocytopenic purpura (TTP).

## 2. Case History

A 36-year-old Caucasian woman, presented to the emergency department with severe asthenia and progressive dyspnea on minimal exertion of one-week duration. She reported having chronic dyspepsia with intermittent diarrhea and weight loss. She denied fever, abdominal pain, headache, or blurred vision. As past medical and surgical history, she was found to have recurrent uncomplicated urinary tract infections and fallopian tubes ligation. She was only on vitamin D supplements. She admitted consuming alcohol on occasions but she never smoked nor used illicit drugs.

Her family history is remarkable for a father who has chronic lymphocytic leukemia under active surveillance and a hypothyroid (Hashimoto's disease) mother.

On admission, the patient was afebrile, hemodynamically stable, eupneic with an oxygen saturation of 98% on room air, conscious, oriented, and cooperative but lack of energy was noted. She appeared pale with noninjected conjunctiva. No ecchymosis, mucosal lesions, or lymphadenopathy were detected. She had tachycardia with normal heart sounds and her lungs were clear. On abdominal examination, she had mild splenomegaly but no hepatomegaly. Neurologic examination showed normal sensation and no motor deficit.

**Figure 1:**
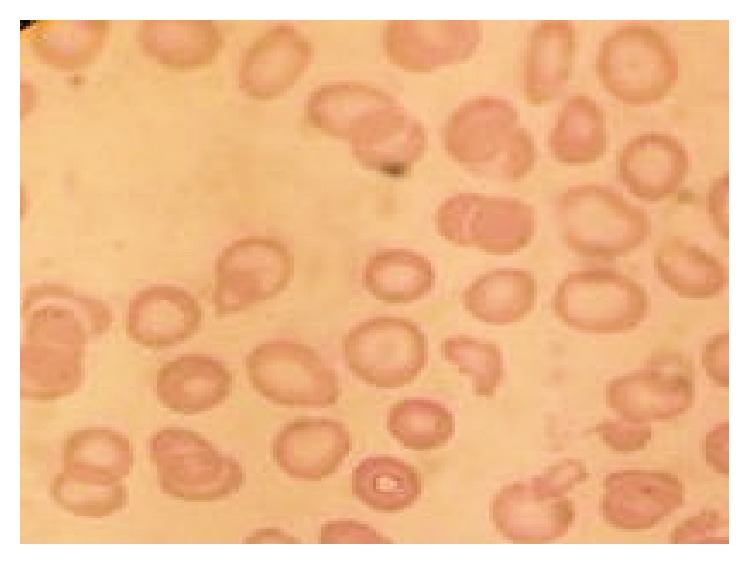
Peripheral blood smear.

The laboratory tests showed hemoglobin 7.8 g/dL, hematocrit 23.5%, MCV 102 fl, WBC 2,800/mm^3^, N: 55% L: 40% M: 4% E: 1%, platelet count 100,000/mm^3^, BUN 9.53 mg/dL, and creatinine 0.65 mg/dL, that electrolytes were normal, that total bilirubin was upper limit of normal range, normal liver enzymes, and that serum LDH was 1828 U/L (Table 1). Direct coombs test was negative and indirect coombs tests at 4 and 37 degrees were negative. Haptoglobin was less than 0.1 g/L and D-dimer 1.43 *μ*g/mL. Prothrombin time, partial thromboplastin time, and serum fibrinogen were normal. Peripheral smear revealed 3% of schistocytes, anisocytosis, and macrocytes (Figure 1).

Thrombotic microangiopathic anemia was suspected. The patient was kept under close surveillance. She had no neurologic symptoms, no headache, and no blurred vision. ADAMST13 activity test was normal. Vitamin B12 dosage was 182.6 pg/mL (normal 200–866 pg/mL), folic acid 13.9 ng/mL (normal 3.5–16.1 ng/mL), reticulocyte count 1.1%, iron 51 *μ*g/dL (normal 60–178 *μ*g/dL), transferrin 255 mg/dL (normal 188–341 mg/dL), saturation 16.5% (normal 20–50%), ferritin 32 ng/mL (normal 10–250 ng/mL), TSH 3.74 *μ*U/mL (normal 0.4–5 *μ*U/mL), and homocysteine level 44 *μ*mol/L (normal 3.36–20.4 *μ*mol/L). The osmotic test was normal and the cold agglutinin negative. Bone marrow core biopsy and aspirate showed hypercellular marrow with erythroid hyperplasia, and the erythroid precursors presented megaloblastic features.

Treatment with 1000 *μ*g of vitamin B12 in intramuscular injections on a daily basis for 10 days resulted in hematological and clinical improvement, along with a decrease in D-dimer level.

**Figure 2:**
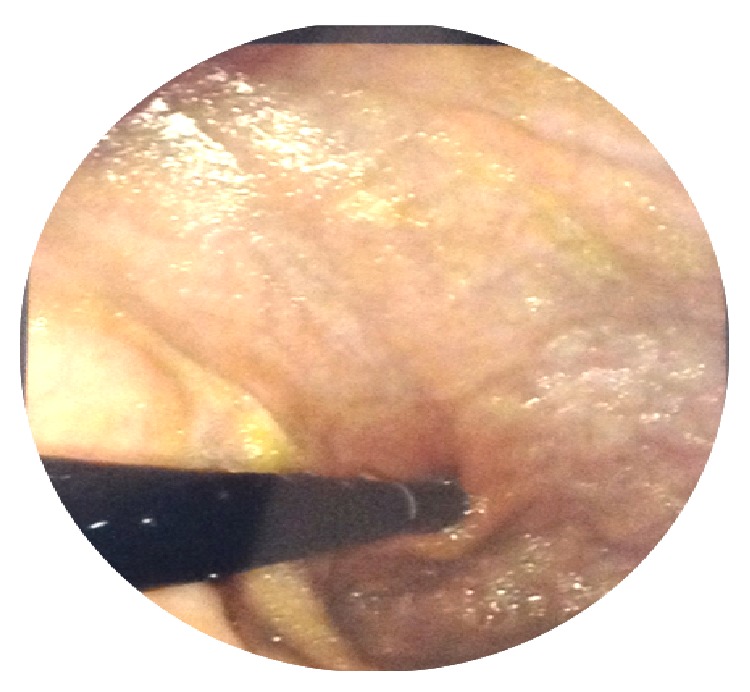
Atrophic gastritis of the fundus.

Gastroscopy (Figure 2) with biopsies revealed severe chronic atrophic gastritis with intestinal metaplasia sparing the antrum. Colonoscopy was normal and did not reveal terminal ileitis. The blood test showed positive intrinsic factor antibodies. The latter test yields a sensitivity and specificity of 37% and 100%, respectively, thus confirming the diagnosis of autoimmune atrophic gastritis (also known as Biermer's disease, pernicious anemia, and Addisonian anemia). At one-month follow-up, our patient was asymptomatic with hemoglobin 12 g/dL, white blood cell count 7,100/mm^3^, platelet count 190,000/mm^3^, and LDH 433 U/L.

## 3. Discussion

The thrombotic microangiopathy syndromes (TMA) are defined by clinical and pathologic characteristics. The clinical features include microangiopathic hemolytic anemia, thrombocytopenia, and organ injury. The pathologic characteristics are vascular damage that is manifested by arteriolar and capillary thrombosis [5, 6]. Thrombotic microangiopathies are infrequent, critical conditions associated with a mortality rate of 10–20% [7]. The differential diagnosis for microangiopathic hemolytic anemia includes disseminated intravascular coagulation (DIC), malignant hypertension, severe preeclampsia/HELLP syndrome, bone metastatic cancers, and prosthetic heart valves. Aside from these conditions, vitamin B12 deficiency in the context of autoimmune atrophic gastritis can mimic TMA.

In humans, there are two enzymatic reactions known to be vitamin B12 dependent. The first one is methylmalonic acid converted to succinyl-coA and the second is homocysteine converted to methionine [4]. Both are primordial for cellular DNA synthesis; consequently, the major organs affected by lack of vitamin B12 are those with rapid cell turnover, such as bone marrow, resulting in macrocytic anemia, ineffective erythropoiesis, and rare life threatening conditions such as pancytopenia, and microangiopathic hemolytic anemia as seen in our patient.

The causes of vitamin B12 deficiency are mainly food cobalamin malabsorption, autoimmune atrophic gastritis, total or partial gastrectomy, gastric bypass, terminal ileal resection, inflammatory bowel disease, tropical sprue, nutritional deficiencies, and certain drugs, such as metformin [4, 8]. As in our case, the cause of vitamin B 12 deficiency is pernicious anemia, an autoimmune gastritis that is supported by the presence of mononuclear cell infiltration into gastric mucosa along with autoantibodies against parietal cells, intrinsic factor and gastric H^+^/K^+^ATPase [3].

We noticed in our case an elevation of D-dimer levels (Table 1). A D-dimer is the degradation product of cross-linked fibrin and it reflects the activation of hemostatic and thrombolytic systems. It may be increased in the following conditions: postsurgical procedures, pregnancy, malignancy/inflammation, trauma, cardiovascular/venous thromboembolic disease, and liver disease. Therefore, deficiency in vitamin B12 results in hyperhomocysteinemia that leads to platelet activation, generation of reactive oxygen species, loss of endothelial system homeostasis, vasoconstriction, increased tissue factor expression, and coagulation activation [9, 10]. We concluded that hyperhomocysteinemia could raise the level of D-dimer through the activation of the coagulation cascade and lead to fragmentation of red blood cells to schistocytes as seen in our patient [10]. Strikingly, we observed the decreased level of schistocytes on peripheral blood was correlated to the decreased level of D-dimer (Table 1).

In 2013, Noël et al. provided clinical and laboratory features that are useful in discriminating between TTP and pseudo-TMA in the emergency department [7]. Moreover, they compared the seven pseudo-TMA patients with other cobalamin deficient patients without schistocytosis, and they found that pernicious anemia was the main cause of vitamin B12 deficiency in pseudo-TMA group as in our patient. Based on this result and the principal role of immune system plus T cells dysregulation in pathophysiology of autoimmune atrophic gastritis [3], we think that it could be a predisposing immunologic mechanism leading to thrombotic microangiopathy in patients with pernicious anemia. Further research and studies are recommended in this domain to confirm the potential role of autoimmunity.

## 4. Conclusion

The core tip of this case is to maintain a high index of suspicion for unusual clinical manifestations of autoimmune atrophic gastritis, vitamin B12 deficiency with schistocytes, and microangiopathic hemolytic anemia, because it may be misdiagnosed with other critical diseases such as thrombotic thrombocytopenic purpura. Thus, it is essential for physicians to be familiar with the various clinical features of pseudothrombotic microangiopathy and to assess the severity of this entity promptly and accurately, seeing as appropriate treatment is simple and effective with good prognosis and prevents unnecessarily invasive therapeutic measures such as plasmapheresis, thereby reducing the morbidity and mortality of this potentially life-threatening condition.

## Figures and Tables

**Table 1:** Laboratory values on admission, one week and at one-month follow-up after admission.

Laboratory variable	Reference range (adult)	On admission	One week after admission	At one-month follow-up
Hemoglobin (g/dL)	12–16	7.8	10	12
Hematocrit (%)	37–47	23.5	29.8	36.6
White cell count (per mm^3^)	4,000–11,000	2,800	4,600	7,100
Platelet count (per mm^3^)	150,000–400,000	100,000	140,000	190,000
Mean corpuscular volume (fl)	75–95	102	97	81
Blood urea nitrogen (mg/dL)	7–22	9.53	16	10.6
Creatinine (mg/dL)	0.5–1.2	0.65	0.72	0.69
Bilirubin (mg/dL)				N/A
Total	0.2–1.5	1.3	0.8	
Direct	0.0–0.3	0	0	
Haptoglobin (g/L)	0.3–2	0.1	N/A	N/A
Lactate dehydrogenase (U/L)	313–618	1828	681	433
INR	0.9–1.2	1	1	N/A
Prothrombin time (seconds)	10–13	10	10	N/A
Activated partial thromboplastin time (seconds)	23–31	24	25	N/A
Schistocytes (%)	0%	3%	0.8%	N/A
D-dimer (*μ*g/mL)	Less than 0.5	1.43	1	N/A
